# Autonomic Dysregulation in Obsessive–Compulsive Disorder: Simultaneous Assessment of Electrodermal, Cardiac, and Oculomotor Responses to Triggering Videos—An Exploratory Study

**DOI:** 10.3390/brainsci16080846

**Published:** 2026-08-09

**Authors:** Galina Portnova, Guzal Khayrullina, Emily Bainbridge, Olga Martynova

**Affiliations:** Institute of Higher Nervous Activity and Neurophysiology of RAS, 5A Butlerova Str., Moscow 117485, Russiaomartynova@ihna.ru (O.M.)

**Keywords:** obsessive–compulsive disorder, heart rate variability, galvanic skin response, eye-tracking, autonomic dysregulation, triggering video

## Abstract

**Highlights:**

**What are the main findings?**

- The OCD group exhibited video-triggered sympathetic hyperactivation, characterized by lower mean NN intervals and a progressive rise in tonic GSR, whereas healthy controls showed greater autonomic flexibility (rapid increases in SDNN and RMSSD) following pleasant videos.
- Individuals with OCD demonstrated faster saccade velocities but a notable decoupling of eye-movement responses from the emotional pleasantness of video content.

**What are the implications of the main findings?**

- Reduced autonomic flexibility—specifically, delayed sympathovagal shifting—may serve as a core, quantifiable feature of OCD, with potential utility for diagnostic assessment.
- Tonic GSR, RMSSD, and saccade velocity are proposed as candidate biomarkers suitable for in-tegration into wearable monitoring systems and biofeedback-based therapeutic interventions.

**Abstract:**

Background: The autonomic nervous system (ANS) in individuals with obsessive–compulsive disorder (OCD) is often characterized by heightened sympathetic tone and reduced parasympathetic flexibility. This exploratory pilot study aimed to provide a detailed, dynamic portrait of autonomic dysregulation. Methods: Heart rate variability (HRV), galvanic skin response (GSR), and oculomotor measures (pupil size, fixations, saccade velocity) were recorded in 31 participants with OCD (obsessive–compulsive disorder) and 46 healthy controls while viewing 18 videos, half of them designed to trigger specific OCD dimensions and half neutral or positive. Results: The OCD group exhibited a state of sympathetic hyperactivation, evidenced by significantly lower mean NN intervals (average interval between normal heartbeats) and a distinct GSR (galvanic skin response) pattern marked by a rising tonic component across the experiment. Healthy controls exhibited increases in SDNN (standard deviation of NN intervals) and RMSSD (root mean square of successive differences) following pleasant videos, whereas this response was less pronounced and emerged later in the OCD group, suggesting reduced autonomic flexibility. Oculomotor data revealed faster saccade velocities during video viewing in the OCD group; however, unlike controls, their eye movements did not correlate with the pleasantness of the content. Conclusions: These results support the theory that individuals with OCD may exhibit greater sympathetic activation and delayed parasympathetic engagement. The findings suggest that autonomic inflexibility may be a core, measurable feature of OCD.

## 1. Introduction

The autonomic nervous system (ANS) serves as a critical interface between cognitive processes and physiological adaptation, with the balance between its sympathetic and parasympathetic branches reflecting an individual’s capacity to respond to and recover from environmental challenges [1]. Autonomic flexibility refers to the dynamic ability of the autonomic nervous system to adapt to internal and external stressors by shifting between sympathetic and parasympathetic dominance [2]. Disruptions in this balance have been consistently implicated in the pathophysiology of various mental disorders, including obsessive–compulsive disorder (OCD) [1]. While previous research has reported heightened sympathetic tone and reduced parasympathetic flexibility in OCD, findings across studies have been inconsistent. Moreover, most studies have relied on resting-state recordings rather than dynamic, stimulus-evoked paradigms. Furthermore, research on autonomic dysregulation in OCD has largely relied on individual or paired physiological measures, for example, HRV and GSR [3], HRV and eye-tracking [4], and GSR alone [5]. Although each of these parameters has shown diagnostic potential, their simultaneous assessment within a single paradigm remains uncommon. Critically, to our knowledge, no study has simultaneously recorded cardiac, electrodermal, and oculomotor responses during symptom-provoking video viewing in individuals with OCD. The present study fills this gap by integrating all three channels to provide a comprehensive characterization of autonomic response patterns.

There are relatively few studies on HRV in OCD populations, and their results are inconsistent. Several studies failed to find significant differences between clinical and control groups [6,7]. A more recent study also did not find group differences in HRV but did observe higher heart rate in OCD participants [8]. Cardiac rhythm, controlled by the ANS, varies according to stress, genetics, and activity levels, making HRV a valuable index of autonomic health [9,10]. A wide range of HRV metrics—such as the average interval between normal heartbeats (NN), the standard deviation of NN intervals (SDNN), and the root mean square of successive differences (RMSSD)—offer nuanced insights into cardiac and psychological well-being [11,12]. Lower SDNN and RMSSD have been associated with increased all-cause mortality and cardiovascular risk [12], while reduced HRV is also observed in anxiety and depression [13,14]. However, findings in OCD populations remain inconsistent: some studies report lower HRV and higher heart rate in OCD patients [15], while others fail to find significant group differences [6,7,8]. This variability underscores the need for further investigation, particularly using dynamic, stimulus-evoked paradigms rather than resting-state recordings alone.

Complementing cardiac measures, galvanic skin response reflects sympathetic sudomotor activity and comprised a slowly changing tonic component (skin conductance level, SCL) and rapid phasic responses (skin conductance response, SCR) to discrete stimuli [16,17,18]. GSR has proven useful in detecting hidden emotional states in vulnerable populations, including individuals with autism, as well as in consumer research [19,20,21,22,23,24]. Importantly, GSR can be combined with other biomarkers, such as pupil diameter, to form a multifaceted overview of mental states [3,25].

Research on GSR in patients with OCD is even more limited. Previous studies demonstrated that OCD patients had significantly lower skin conductance levels and responses compared to patients with generalized anxiety disorder (GAD), panic disorder (PD), and healthy controls across baseline, stress, and recovery phases [5]. Although OCD patients showed some reactivity to stress (baseline vs. stress), they exhibited significant difficulty returning to pre-stress resting levels. GSR values in OCD fell within the normal range but differed from healthy controls specifically in recovery capacity [5]. Other researchers reported that OCD patients showed significantly lower GSR baseline values compared to GAD and PD groups, while OCD and major depressive episode (MDE) patients had similarly low electrodermal activity [26]. Furthermore, OCD patients showed minimal GSR increase during mental arithmetic stress, and their GSR did not return to pre-stress levels significantly afterwards, unlike patients with generalized anxiety disorder (GAD) and panic disorder (PD) [26]. Importantly, GSR can serve as a physiological biomarker of treatment response, a finding potentially applicable to OCD [8,27].

Another set of parameters related to ANS regulation includes pupil and oculomotor responses. Oculomotor measures, particularly saccade velocity, offer another window into the autonomic nervous system. Average and peak saccade velocity are sensitive to alertness, task engagement, and the motivational value of stimuli [28,29,30]. High-value images elicit faster saccades [30], and video game players show elevated peak velocities [31]. Importantly, psychiatric disorders such as schizophrenia, bipolar disorder, anxiety, and ADHD are associated with altered saccadic performance [32]; yet saccade velocity in OCD remains strikingly understudied.

Previous studies found that OCD patients had consistently larger pupil areas compared to healthy controls, which was interpreted as indicating heightened sympathetic tone at baseline. Furthermore, healthy volunteers showed adaptive decreases in pupil area across repeated task blocks, whereas OCD patients exhibited reduced adaptation, and this lack of adaptation correlated with greater symptom severity on the Y-BOCS [4]. In contrast, Pöhlchen [33] examined pupil dilation during a fear conditioning paradigm and found no differences between OCD patients and healthy controls in pupillary responses during fear acquisition or extinction, suggesting that certain phasic pupil responses may remain intact in OCD. Regarding saccade speed, reflexive orienting appears largely intact in OCD, but volitional responses may be slowed. Patients with OCD do not show faster saccadic orienting (vigilance bias) toward OCD-relevant stimuli compared to healthy controls [34,35]. In fact, both groups orient more quickly to emotionally salient pictures regardless of diagnosis. Peak saccade velocity is generally normal in OCD, although some studies report slower velocities for large-amplitude saccades [36]. Saccade frequency is often increased in OCD, manifesting as a higher number of anticipatory and intrusive saccades during fixation [37]. Bradley [35] found that symptom severity predicted more frequent fixations (i.e., more saccadic returns). Other researchers reported more unwanted saccades during slow smooth pursuit and reduced fixation stability, particularly in patients receiving antipsychotic medication [38]. Lencer demonstrated impaired smooth pursuit maintenance (reduced steady-state velocity), suggesting frontal network dysfunction, especially in the pursuit region of the frontal eye fields [39].

Given the fragmented literature and the lack of simultaneous assessment across multiple autonomic channels, the present study aimed to characterize autonomic dysregulation in OCD using a comprehensive, dynamic approach, moving beyond static descriptions toward a more nuanced understanding of reduced autonomic adaptability as a potential core feature of OCD.

We simultaneously recorded HRV, GSR, and eye movements (including pupil size, fixations, and saccade velocity) in individuals with OCD and healthy controls while they viewed a series of emotionally evocative videos—half of which were designed to trigger specific OCD dimensions (aggression, checking, contamination, symmetry) and half neutral or pleasant. We hypothesized that individuals with OCD would exhibit greater sympathetic activation at baseline and during symptom-provoking stimuli, as well as demonstrate an imbalance in parasympathetic and sympathetic dynamics while watching the videos. By integrating these measures, we sought to generate hypotheses regarding dynamic autonomic patterns that may characterize OCD, with the understanding that these findings will require replication in larger, methodologically controlled studies.

## 2. Methods

### 2.1. Participants

The experiment included 46 healthy volunteers (mean age 27.4 ± 8.6 years, range 17–45, 28 females) and 31 volunteers with OCD symptoms (mean age 28.3 ± 7.2 years, range 18–44, 22 females). Before participation, all volunteers completed online screening using the Beck Depression Inventory (BDI), State-Trait Anxiety Inventory (STAI), Barratt Impulsiveness Scale (BIS-11), and a general medical questionnaire (head trauma, insomnia, medications). The OCD group additionally completed the Yale-Brown Obsessive Compulsive Scale (Y-BOCS) and underwent a clinical interview by a clinical psychologist (G.K.) and a medical doctor (G.P.). Healthy volunteers were excluded if test scores indicated significant psychological distress. Medication use in the OCD group (neuroleptics, antidepressants, monotherapy, combination, or no therapy) was recorded and statistically controlled. Full results of the screening can be seen in Table 1. Among the 31 patients with OCD in this cohort, half (16 patients) were actively taking psychiatric medication, while the other 15 patients were unmedicated. Of those receiving treatment, the most commonly prescribed class was SSRIs, used by 10 patients, often as a first-line therapy. A smaller number of patients were on other drug classes: 6 patients were treated with SGAs (second-generation antipsychotics), 4 patients received SNRIs, 4 patients were on AEDs (antiepileptic drugs), and only 2 patients were prescribed TCAs (tricyclic antidepressants). See details in Supplementary (Table S1).

All participants provided written informed consent.

### 2.2. Ethical Approval

The study was approved by the Institutional Ethics Committee of the Institute of Higher Nervous Activity and Neurophysiology of RAS (Protocol No. 4, dated 15 March 2023). The study was conducted in accordance with the Declaration of Helsinki. In compliance with ethical guidelines, all participants provided written informed consent before the experiment, after being thoroughly briefed on the study procedures (including exposure to unpleasant videos) and on their right to discontinue participation at any time without explanation. Given the potentially distressing nature of some videos (e.g., violent content, contamination triggers), participants were monitored throughout the session by a clinical psychologist and a medical doctor. A debriefing session was provided after the experiment, and any participant reporting significant distress was offered appropriate support.

### 2.3. Stimuli

All video stimuli were 20 s in duration, silent, and presented at 880 × 660 pixels, 29.97 fps, with a bitrate of 2722 kbps. Eighteen videos were divided into four OCD subtypes (aggression, checking, contamination, symmetry), each containing two OCD-triggering and two neutral/positive videos (showing ideal outcomes). Two additional kitten videos served as controls. Example stimuli: a symmetrical mosaic being cleaned (positive), a woman threatened at knifepoint (aggression OCD), a house fire (checking OCD), cockroaches (contamination OCD), asymmetric tiling (symmetry OCD). Stimuli were sourced from open internet resources and are freely available for research use; copyright permissions were verified by the authors. The video order was fixed across participants (see Supplementary Materials for full sequence). After each video, participants rated emotional valence and intensity on a scale of 1–9.

Video stimuli were selected based on a pilot validation study conducted prior to the main experiment. Thirty-seven independent respondents assessed a pool of 29 videos for valence, arousal, specific emotions (joy, fear, anger, disgust, sadness, surprise), and relevance to OCD symptom dimensions. Based on these ratings and inter-rater agreement, 18 videos were selected for the main experiment, comprising four OCD subtypes (aggression, checking, contamination, symmetry), each containing two OCD-triggering and two neutral/positive videos, with two additional kitten videos serving as controls. The final stimulus set showed high inter-rater reliability for valence (Cronbach’s α = 0.89) and arousal (Cronbach’s α = 0.87), with consistency scores ranging from 0.25 to 0.92. Videos with low consistency were excluded, and the selected set demonstrated clear differentiation between triggering and control stimuli across the relevant emotional dimensions.

The video order was fixed across participants (see Supplementary Materials for the full sequence). To mitigate potential order effects, we arranged the videos in a pseudorandomized manner, ensuring that no two trigger videos from the same category were presented consecutively and that pleasant and unpleasant videos were balanced over time throughout the session. However, this fixed order remains a limitation of this exploratory pilot study and should be taken into account when interpreting temporal dynamics. The details are presented in Table S2.

### 2.4. Physiological Recordings

Galvanic skin response (GSR) was measured using two GSR sensors (Medical Computer Systems Ltd., Moscow, Russia) placed on the second knuckle of the first and third fingers of the dominant hand. The blood pulse sensor (Becker Meditec, Karlsruhe, Germany) was placed on the middle finger of the same hand. Both signals were recorded using an actiCHamp Plus amplifier (Brain Products GmbH, Gilching, Germany) and BrainVision Recorder (version 1.23.0001). Markers were synchronized to video onset and keypress responses via Experiment Builder (SR Research Ltd., Ottawa, ON, Canada).

### 2.5. Eyetracker

Eye movements were recorded using an EyeLink Portable Duo eye-tracker (SR Research Ltd., Ottawa, ON, Canada; sampling rate 500 Hz) in a dimly lit (3.4 lux), sound-proofed room. Participants were seated 70 cm from a 70 cm monitor (Asus Vision XG248q, 240 Hz, ASUSTeK Computer Inc., Taipei City, Taiwan) with their chins on a chinrest. Nine-point calibration was performed before the video block.

### 2.6. Data Preprocessing and Analysis

HRV: ECG data were recorded at a sampling rate of 500 Hz using actiCHamp Plus amplifier (Brain Products GmbH, Gilching, Germany). Offline preprocessing was performed using Kubios HRV (Standard version v3.5.0, Kubios Oy, Finland). Raw ECG signals were band-pass filtered at 0.5–40 Hz with a 50 Hz notch filter to remove power line interference. R-peaks were detected automatically using the Kubios adaptive threshold algorithm and verified manually by a trained researcher. Ectopic beats were identified as intervals differing by more than 30% from the preceding five normal-to-normal (NN) intervals and were corrected using cubic spline interpolation. Epochs containing more than 5% ectopic beats were excluded from further analysis (less than 3% of all epochs were discarded). Artifacts were also visually inspected and removed where necessary. HRV parameters—mean NN interval, SDNN, and RMSSD—were computed for each 20 s epoch corresponding to each video presentation and inter-stimulus pause, as well as for the 2 min initial resting period.

The continuous recording was segmented into non-overlapping 20 s epochs aligned with each video presentation (18 videos × 20 s) and each inter-stimulus pause (17 pauses × 20 s), yielding 35 experimental epochs in addition to the initial 2 min resting state. Epoch boundaries were synchronized to video onset and offset via trigger markers recorded in BrainVision Recorder and verified using Experiment Builder (SR Research Ltd., Ottawa, ON, Canada).

GSR: GSR signals were recorded using two Ag/AgCl electrodes placed on the second knuckle of the first and third fingers of the dominant hand. The GSR sensor was part of the actiCHamp Plus amplifier system (Brain Products GmbH, Gilching, Germany). Signals were sampled at 500 Hz and preprocessed using a low-pass filter at 5 Hz. Offline decomposition into tonic (skin conductance level, SCL) and phasic (skin conductance response, SCR) components was performed using Ledalab (version 3.4.9, University of Würzburg, Germany) with continuous decomposition analysis (CDA). CDA employs a non-negative deconvolution approach to separate the slowly varying tonic component from rapid phasic events. Phasic responses were scored as peak amplitude within a 1–5 s window following stimulus onset, with a minimum response criterion of 0.01 µS. Baseline SCL was defined as the mean tonic level during the 10 s immediately preceding each video. Artifacts (e.g., movement-related spikes) were identified by visual inspection and removed prior to decomposition.

Eye tracking: Eye movements were recorded using an EyeLink Portable Duo eye-tracker (SR Research Ltd., Ottawa, ON, Canada) at a sampling rate of 500 Hz. Fixations were identified using the EyeLink default algorithm (velocity threshold: 30°/s; acceleration threshold: 8000°/s^2^; motion threshold: 0.1°). Saccades were detected using the same algorithm; peak and average saccade velocity, as well as average saccade amplitude, were extracted for each epoch. Blinks were detected based on pupil diameter falling below 2 standard deviations of the mean and were excluded from fixation and pupil analyses. Only trials with a minimum of 75% valid data (i.e., less than 25% data loss due to blinks or tracking loss) were retained; this resulted in the exclusion of approximately 2% of epochs. For pupil size analysis, artifacts were identified and interpolated using linear interpolation for gaps shorter than 200 ms; longer gaps were excluded. Pupil size was averaged per epoch after artifact removal.

### 2.7. Statistical Analysis

Analyses were conducted using Statistica 13 (StatSoft). Significance was set at *p* < 0.05. Two-way mixed ANOVAs (Group × Time) were performed for each measure, with Bonferroni post hoc corrections unless noted. Effect sizes were assessed using partial η^2^. Spearman correlations examined relationships between subjective ratings and physiological measures. For the ANCOVA analysis of antidepressants, medication status was coded dichotomously: patients not taking antidepressants were coded as 0, and those taking antidepressants were coded as 1. However, given that many medicated patients received combination therapy, we employed a multi-indicator approach rather than treating pharmacotherapy as a single categorical variable. To account for the potential influence of specific psychotropic classes, we additionally coded the overall medication regimen on an ordinal scale based on the number of distinct drug classes used: 0 for no medication, 1 for one class, 2 for two classes, and 3 for three or more classes. No significant effects were found. The fixed video order was acknowledged as a limitation, and a supplementary Spearman correlation between experimental period and group differences in HRV was conducted to assess temporal confounding.

Sample size was determined a priori using G*Power 3.1. Assuming a medium-to-large effect size (Cohen’s f = 0.25), α = 0.05, and power = 0.80, the required total sample size was estimated at 52 participants. We recruited 31 OCD patients and 46 healthy controls, which was adequate for this exploratory pilot study, though we acknowledge the OCD group sample size as a limitation.

Missing data arose primarily from ECG artifacts (less than 3% of epochs), eye-tracking data loss (approximately 2% of epochs), and occasional GSR recording issues. Missing data were handled by listwise deletion for the affected epochs only, meaning that each statistical analysis was performed on the maximum available data for that specific parameter. No participant was excluded entirely due to missing data.

To assess whether the fixed video order confounded our results, we performed a supplementary Spearman rank-order correlation between experimental period (from initial rest to final video, numbered 1 to 36) and the *p*-values of post hoc group differences for each state. The correlations were only significant for HRV data.

Given the exploratory nature of this study and the fixed stimulus order, all findings should be interpreted as preliminary and hypothesis-generating.

## 3. Results

### 3.1. Volunteer Screening Stimuli Assessments

OCD-symptomatic participants demonstrated significantly higher arousal levels in response to most videos compared to the control group (F(1, 71) = 12.77, *p* = 0.001, partial η^2^ = 0.15). Post hoc analysis revealed that this difference was significant for videos 2, 3, 4, 5, 6, 9, 10, 13, 14, 16, 17, and 18 (*p* < 0.05). The results are presented in Table 2.

Although the overall perceived pleasantness of the stimuli did not differ between groups, a significant stimulus-by-group interaction was observed (F(17, 1207) = 2.94, *p* < 0.001, partial η^2^ = 0.07). This interaction indicated that OCD-symptomatic participants exhibited an intensified evaluation pattern, rating pleasant videos as more pleasant and unpleasant videos as more unpleasant than the control participants. These differences reached significance for videos 3, 4, 6, 9, 10, 12, 14, 16, and 18 (*p* < 0.05).

### 3.2. Heart Rate Variability

The mean NN (1/HR) was significantly higher in the control group compared to the OCD-symptomatic group (F(1, 75) = 5.401, *p* = 0.023, partial eta-squared = 0.07). The group differences varied depending on the video and resting state (F(35, 2485) = 1.444, *p* = 0.045) and decreased significantly during the study; however, given the fixed stimulus order, these temporal patterns should be interpreted with caution. Spearman’s rank-order correlation between experimental period (from initial rest to final video, numbered 1 to 36) and the *p*-values of post hoc group differences for each state showed significant positive correlation (r = 0.57, *p* = 0.0002) was observed for HRV, suggesting that group differences in cardiac measures became more pronounced over time, which may reflect cumulative anxiety or sensitization. This finding is acknowledged as a limitation.

No significant main group effect or Group × Time interaction was found for SDNN or RMSSD. However, a significant main effect of time was observed for both parameters (SDNN: F(35, 2485) = 1.47, *p* = 0.038; RMSSD: F(35, 2485) = 2.60, *p* < 0.001). Post hoc comparisons with Bonferroni correction revealed that healthy controls showed a significant increase in both SDNN and RMSSD following Video 4 (*p* = 0.003) and Videos 9–10 (*p* < 0.006). In contrast, the OCD group exhibited increased SDNN and RMSSD only toward the end of the experiment (*p* < 0.002), while the control group showed a significant change only in RMSSD at that later time point (*p* = 0.02).

**Figure 1:**
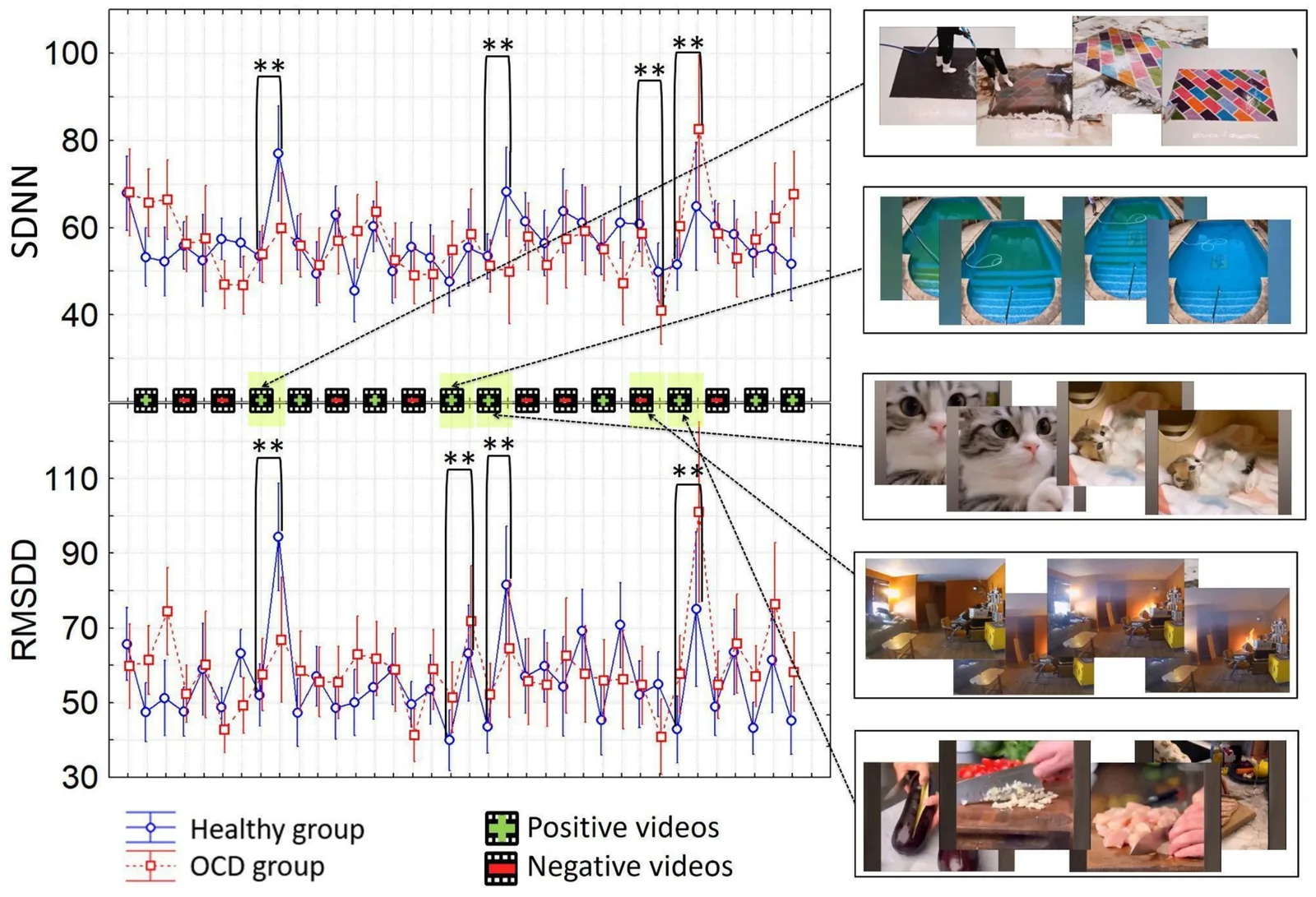
Dynamic changes in heart rate variability (HRV) parameters (RMSSD and SDNN) in patients with OCD and healthy controls during video viewing and inter-stimulus pauses. Data are presented across the experimental timeline. Arrows indicate the position of video stimuli depicted on the right side of the figure. Video sequence and valence are indicated (green—positive/neutral; red—negative). Brackets and asterisks (**—*p* < 0.01) mark significant differences between specific videos and their subsequent pauses. Green ‘+’ indicates positive/neutral valence; red ‘−’ indicates negative valence. The video order is fixed as shown.

Thus, the temporal pattern of SDNN and RMSSD increases differed between groups, with healthy controls showing earlier increases following positive stimuli, while the OCD group showed delayed increases. The results are depicted in Figure 1.

Furthermore, when viewing Video 14 (depicting the gradual start of an apartment fire), OCD-symptomatic participants showed a significant decrease in SDNN (*p* = 0.004) and, to a lesser extent, RMSSD (*p* = 0.04). These differences were not significant in the healthy volunteer group.

### 3.3. Galvanic Skin Response

The videos induced changes in both the phasic and tonic components of the galvanic skin response (GSR). In both groups, a gradual rise in the tonic component began with the first video. However, immediately after the first video, the increase in the tonic component was significantly greater in the control group compared to in OCD-symptomatic participants.

A subsequent decrease in the tonic component was observed starting from Video 4 (the control video featuring gradual carpet cleaning) specifically in healthy participants. In response to this video, OCD-symptomatic participants also exhibited a significantly larger phasic GSR reaction compared to the healthy volunteers.

Video 8, which depicted a dirty apartment with cockroaches, changed the GSR dynamics and triggered a significant increase in the tonic component in OCD-symptomatic participants compared to the control group. A similar pattern was observed for the phasic component, and this effect persisted during the subsequent pause and into the following video (Video 9).

**Figure 2:**
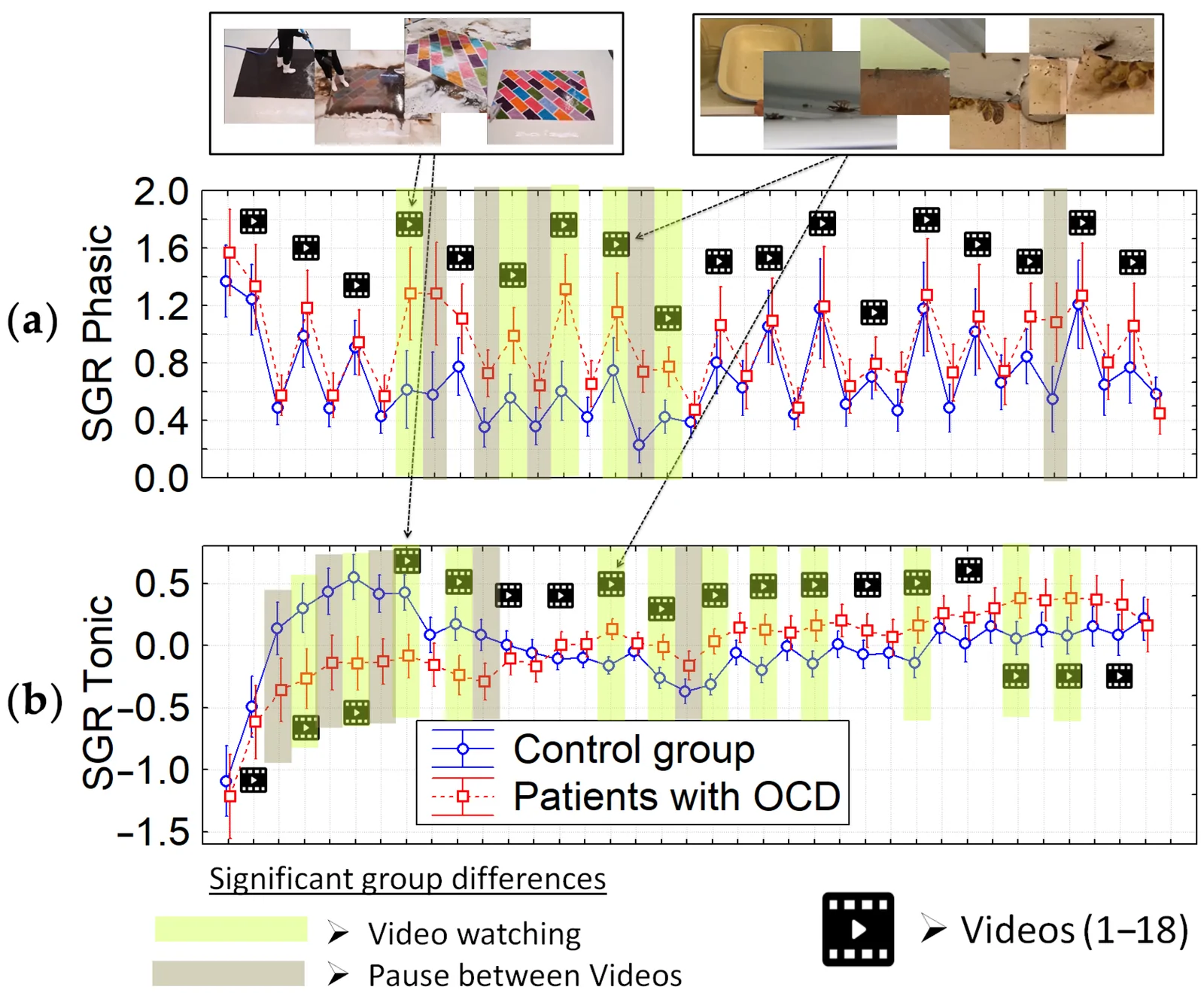
Changes in galvanic skin response (GSR) over the course of the experiment. (**a**) Phasic GSR component during rest, video viewing, and inter-video pauses; (**b**) tonic GSR component during rest, video viewing, and inter-video pauses. Yellow rectangles mark significant differences between groups during video viewing; grey rectangles mark significant differences during inter-video pauses (Bonferroni-corrected post hoc, *p* < 0.05).

Following the viewing of this unpleasant video, the group differences in the tonic component reversed: the GSR tonic activity became significantly more pronounced in OCD-symptomatic participants than in the control group. The progressive increase in tonic GSR in the OCD group may reflect cumulative anxiety; however, the fixed video order prevents us from disentangling stimulus-specific effects from temporal confounds. The results are presented in Figure 2.

### 3.4. Fixations and Pupil Size

**Figure 3:**
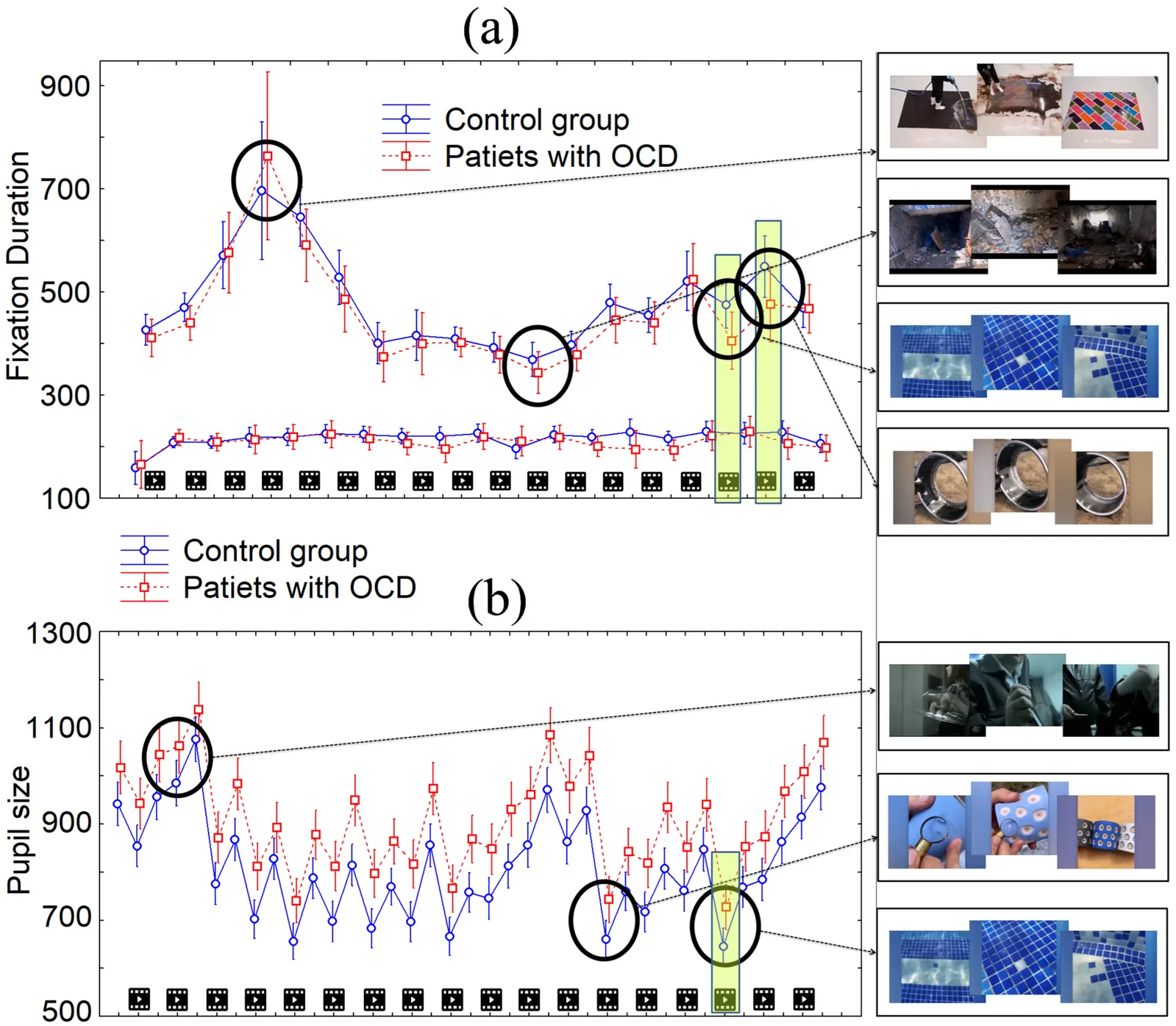
(**a**) Dynamic changes in fixation duration (ms) measured during the resting state, during each of the 18 videos, and during the inter-video pauses. (**b**) Dynamic changes in pupil size measured during the resting state, during each of the 18 videos, and during the inter-video pauses. Yellow rectangles indicate significant intergroup differences for each experimental condition (Bonferroni correction for multiple comparisons, *p* < 0.05).

The average fixation duration and pupil size showed no overall group differences but varied significantly by video. As depicted in Figure 3a, fixation duration increased until Video 4 and then decreased to its lowest during Video 11 (abandoned underpass with litter), increasing again after that in both groups (main stimuli effect F(17, 1275) = 28.249, *p* < 0.001, partial η^2^ = 0.274). Significant group differences were found only for Video 16 and Video 17 (both assessed as pleasant): OCD-symptomatic participants had significantly lower fixation duration during these videos.

The pupil size was higher in OCD-symptomatic participants as a trend (*p* = 0.135), however the pupil size dynamics were similar for both groups (main stimuli effect F(36, 2700) = 118.60, *p* = 0.0000, partial η^2^ = 0.613). A significant group difference was shown only for Video 16 (*p* = 0.031, after LSD post hoc correction), which had the lowest pupil size value of all the videos (Figure 3b).

### 3.5. Saccade Velocity

Peak saccade velocity did not differ between groups during the resting state or inter-stimulus pauses (*p* = 0.163). However, a significant group effect emerged during video viewing (F(1, 75) = 7.799, *p* = 0.007, partial η^2^ = 0.094), indicating that individuals with OCD exhibited heightened oculomotor reactivity specifically when engaged with video content. Similarly, average saccade velocity was significantly higher in the OCD group during video watching (F(1, 75) = 10.584, *p* = 0.002, partial η^2^ = 0.123) but did not differ at rest (*p* = 0.242).

**Figure 4:**
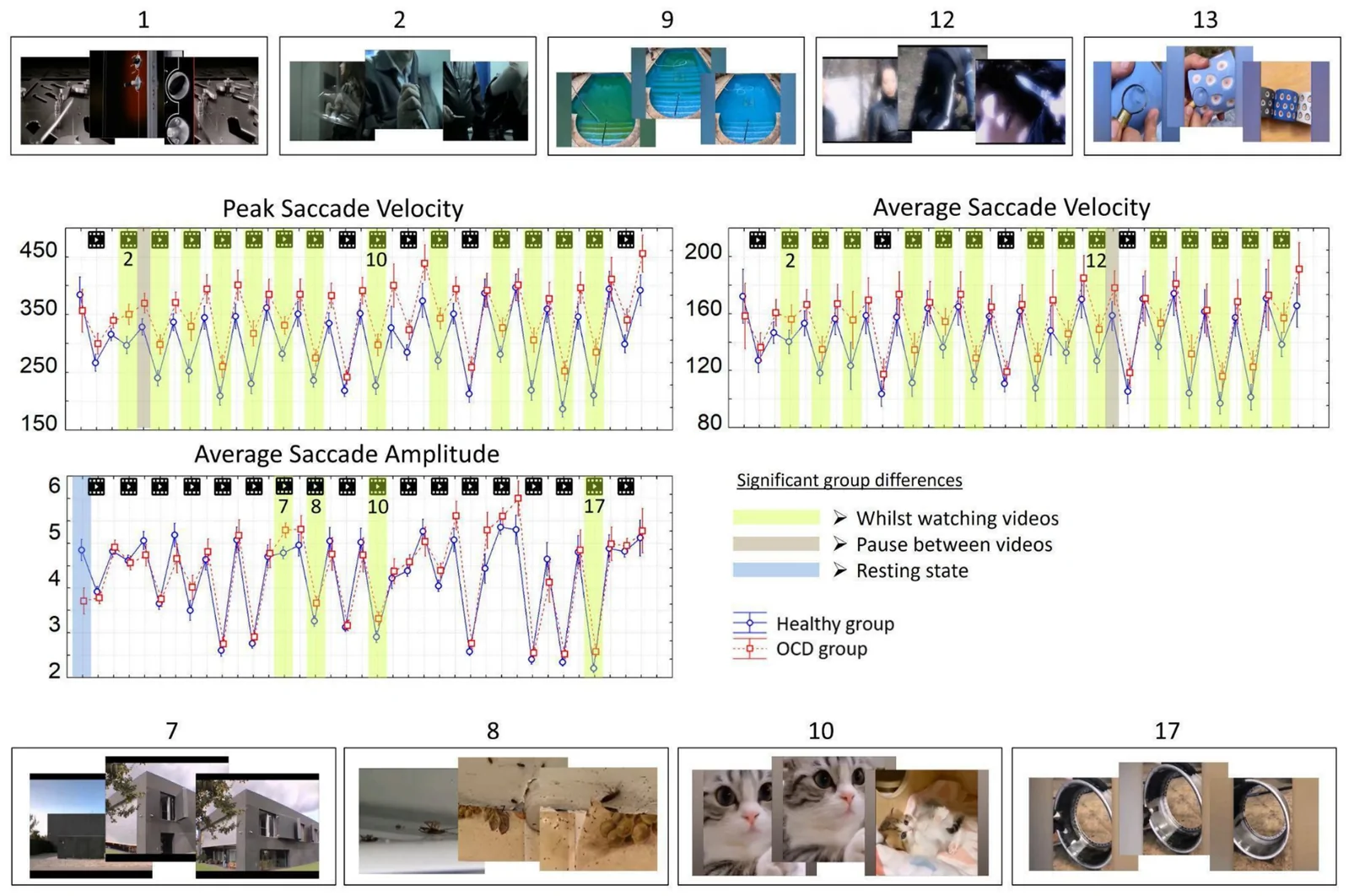
Dynamic changes in saccade kinematics across the experimental session. Time courses of peak saccade velocity (PSV), average saccade velocity (ASV), and average saccade amplitude (ASA) in patients with OCD and healthy controls during the initial rest, video viewing periods, and inter-stimulus pauses. Colored rectangles highlight significant intergroup differences (Bonferroni-corrected, *p* < 0.05) during video viewing (yellow), pauses (grey), and the initial rest (blue). Numerical labels above and below the timeline correspond to the video stimulus number.

In summary, both measures of saccade velocity were higher in the OCD-symptomatic group compared to the control group during video perception. This suggests that the observed differences are stimulus-evoked rather than tonic. The results are depicted in Figure 4.

A main effects analysis showed that average saccade amplitude did not differ significantly over the course of the video or pause periods. However, it was significantly influenced by video type and resting state.

### 3.6. Correlation Analysis

During video viewing, subjective arousal ratings showed a negative correlation with RMSSD levels in both the OCD group and healthy controls. Although the correlation reached nominal significance in the OCD group (ρ = −0.499, *p* = 0.049), it did not withstand correction for multiple comparisons. However, significant correlations unique to the healthy control group were also found. These included a negative correlation between SDNN and arousal (*p* = 0.01), where higher arousal corresponded to lower SDNN, and positive correlations between video valence and both saccade velocity (*p* < 0.041) and amplitude (*p* < 0.031), where higher valence was associated with increased saccade parameters.

In healthy controls, saccade parameters—peak velocity (ρ = 0.507, *p* = 0.032), average velocity (ρ = 0.485, *p* = 0.041), and average amplitude (ρ = 0.510, *p* = 0.031)—correlated positively with video pleasantness. In the OCD group, no such correlations were observed between saccade velocity and subjective ratings.

## 4. Discussion

The temporal pattern of SDNN and RMSSD increases showed descriptive differences between groups. Healthy controls showed increases following positive videos (Video 4 and Videos 9–10), whereas the OCD group exhibited increases only later in the session. This pattern is consistent with a possible delay in parasympathetic engagement in OCD; however, these findings are preliminary and should be interpreted as hypothesis-generating rather than definitive. However, this interpretation is tempered by two important considerations. First, the Group × Time interaction was not significant, meaning the observed differences may not reflect true differential returning to pre-stress levels between groups. Second, the fixed order of video presentation and medication use in some participants introduce potential confounds. Accordingly, these findings should be viewed as preliminary and hypothesis-generating rather than definitive evidence of delayed parasympathetic reactivation in OCD.

Nevertheless, the convergence of findings across three physiological systems strengthens the overall inference. A more adaptive autonomic nervous system (ANS) indicates balance between its sympathetic and parasympathetic branches, and disturbances in this balance have been associated with the development of various mental disorders, including obsessive–compulsive disorder (OCD) [1]. The present study used both positive and negative video stimuli to investigate autonomic regulation in OCD across three complementary physiological systems: cardiac, electrodermal, and oculomotor. Previous studies have found that individuals with OCD show greater sympathetic activation than healthy individuals, both at rest and when faced with aversive stimuli [4,8,15,40]. Our HRV results corroborate these findings, showing significantly lower mean NN (i.e., higher heart rate) in the OCD group. This elevated heart rate may reflect perceived threat from OCD-triggering videos, leading to ANS activation to mediate anxiety. The significantly higher NN observed in the control group indicates a calmer baseline state and better stress adaptation [41]. Conversely, lower NN in the OCD group suggests increased sympathetic activity typical of stressed and anxious states, as well as general maladaptation to environmental stress [4,42,43].

No main group effect emerged for SDNN or RMSSD. However, the control group showed higher SDNN and RMSSD after positive stimuli, indicating greater adaptability and a faster return to pre-stress levels after acute stress. The OCD group exhibited the same effect only toward the end of the experiment. Notably, a high peak in both parameters occurred in the control group after Video 4 (carpet cleaning), after which responses stabilized, whereas the OCD group only showed this increase late in the session. These findings align with prior reports that individuals with OCD have diminished parasympathetic tone and require more time to activate the parasympathetic system and relax from a state of heightened anxiety [8,44]. Overall, the HRV results point to greater sympathetic stress reactivity in OCD, higher baseline excitability, psychological instability, and slower neural adaptation after stress [4].

Turning to electrodermal activity, ANS responses including GSR increase with stress and anxiety [45]. Participants with OCD exhibited a steady increase in tonic GSR throughout the study, whereas healthy controls showed an initial sharp peak after the third video, which then declined and remained lower than the OCD group from the seventh video onward. This pattern further indicates heightened sympathetic response and sustained arousal in the OCD group, suggesting that watching the videos induced progressively rising anxiety. Controls again demonstrated superior regulation of their internal state after an initial arousal spike. Nevertheless, the fixed video order may have confounded these results; the most potent anxiety triggers may have appeared later in the sequence. Future studies should counterbalance video order across participants to rule out order effects. Significant phasic GSR differences between groups were most frequent between the fourth and ninth videos, where the phasic component remained significantly higher in the OCD group. This period coincided with the transition during which tonic GSR in the OCD group rose from being significantly lower than in controls to significantly higher. These results illustrate how the more reactive phasic component can influence the slower-changing tonic GSR [46].

Regarding oculomotor measures, few significant group differences emerged for fixation duration or pupil size, although pupil diameter was consistently higher in the OCD group at a trend level. During Video 16 (asymmetrical cake cutting), OCD participants showed significantly lower fixation duration and significantly higher pupil size than controls. Fixation duration remained higher in the OCD group during Video 17 (positive wheel-rim cleaning video). These findings provide further evidence that individuals with OCD have reduced ability to adapt to acute stress [4]. More striking differences appeared in saccade velocity. Peak saccade velocity was higher in the OCD group for the majority of videos, including all negative videos except Video 11 (the dirty underpass). Average saccade velocity was also higher in the OCD group for all negative videos and six positive videos. Collectively, all eye-movement metrics suggest emotional hyperarousal and increased sympathetic activation in the OCD group. It is important to note that the elevated saccade velocities in the OCD group were observed exclusively during video viewing and not at rest or during inter-stimulus pauses. This pattern indicates that the oculomotor differences reflect heightened reactivity to emotionally salient stimuli rather than sustained baseline hyperarousal. Our finding that saccade velocity differences are stimulus-evoked rather than tonic is consistent with prior work demonstrated that saccade velocity increases with the motivational value of visual stimuli, and that saccade velocity is sensitive to arousal fluctuations during naturalistic tasks [29,30]. Thus, our results align with the view that OCD is characterized by heightened reactivity to emotionally salient stimuli rather than a fixed autonomic trait. To examine the potential influence of stimulus order, we conducted a supplementary Spearman correlation between experimental period and the magnitude of group differences. For saccade velocity, the pattern of group differences did not show a monotonic increase over time; instead, differences were distributed across the session and were more pronounced for negatively valenced stimuli. This argues against a simple order-driven interpretation. Nevertheless, given the fixed order of stimulus presentation, we cannot entirely exclude the possibility that temporal factors contributed to these effects.

The exploratory correlation analysis revealed that, in both groups, lower subjective arousal ratings were associated with higher RMSSD. This association was nominally significant in the OCD group (*p* = 0.049) but did not survive correction for multiple comparisons. Given that this was an exploratory analysis, we report uncorrected *p*-values with the understanding that such findings are hypothesis-generating and require replication. This pattern is consistent with the well-established link between subjective emotional arousal and parasympathetic activity: heightened arousal is typically associated with attenuated heart rate variability, reflecting reduced parasympathetic influence on the heart [1,3,10,47]. Nevertheless, given the exploratory nature of this analysis, this finding should be interpreted with caution until replicated in independent samples.

A second correlational finding also merits attention. In healthy controls, video pleasantness correlated positively with saccade velocity and amplitude (FDR-corrected, q < 0.10), whereas no such association was observed in the OCD group. Previous eye-tracking studies have demonstrated that higher pleasantness or positive valence is associated with increased saccade velocity and amplitude [29,48]. The absence of this association in the OCD group is noteworthy and may reflect a decoupling of oculomotor responses from positive emotional content, consistent with a negativity bias in automatic attentional processes. Given that greater saccade velocity has also been observed in response to high-value visual stimuli [30], it is plausible that individuals with OCD assigned greater salience to negative stimuli—perceiving them as threatening and responding with anxiety—whereas healthy controls showed stronger physiological and oculomotor responses to positive stimuli, suggesting better regulatory capacity. This impaired ability to dynamically shift autonomic and attentional states in response to changing environmental demands may also contribute to the difficulties in fear extinction commonly observed in OCD [49].

From a clinical and translational perspective these findings have direct relevance to wearable devices and biofeedback interventions. The delayed parasympathetic reactivation in OCD, indexed by blunted SDNN and RMSSD responses to positive stimuli, suggests that real-time HRV biofeedback could help patients learn to engage parasympathetic relaxation more quickly. Similarly, tonic GSR emerged as a potential biomarker for sustained arousal; wearable sensors that detect rising tonic GSR could alert patients or clinicians to impending anxiety escalations before they become clinically significant. Finally, the decoupling of saccade velocity from positive valence raises the possibility that eye-tracking metrics might serve as objective indicators of treatment response, particularly for interventions aimed at restoring attentional flexibility. As an exploratory pilot study with a fixed stimulus order, our findings are intended to generate hypotheses for future investigation rather than to establish causal relationships. This design is a primary interpretive concern: the progressive increase in tonic GSR and the delayed HRV responses observed in the OCD group could reflect sequence effects, habituation, or fatigue just as readily as group-specific autonomic dynamics. Moreover, our correlational analyses were exploratory and not adjusted for multiple comparisons, so they are best treated as hypothesis-generating rather than confirmatory. Medication status in the OCD group, though statistically accounted for, may have also modulated physiological reactivity in ways we could not fully disentangle. Taken together, these caveats underscore the preliminary nature of our conclusions and highlight the need for replication in counterbalanced designs with larger samples.

## 5. Limitations

The fixed order of video presentation limits causal interpretation of temporal dynamics, and future studies should counterbalance stimulus order to disentangle content-driven effects from habituation or fatigue. Medication use in the OCD group, though statistically controlled, warrants replication in an unmedicated sample. Additionally, the relatively small OCD sample (*n* = 31) constrained statistical power and precluded rigorous correction for multiple comparisons; therefore, our correlational analyses remain exploratory, and the reported associations—particularly those with marginal *p*-values—should be interpreted cautiously until replicated in larger, independent samples. It should also be noted that our conclusions regarding impaired parasympathetic flexibility are tempered by the absence of a significant Group × Time interaction for SDNN and RMSSD.

## 6. Conclusions

Our preliminary findings support heightened sympathetic activation in OCD during symptom-provoking stimulation, evidenced by lower mean NN intervals and sustained tonic GSR. The temporal profiles of SDNN and RMSSD suggest differences in parasympathetic engagement, with healthy controls showing earlier increases to positive stimuli. Although this is consistent with reduced autonomic flexibility, the fixed stimulus order and the absence of a significant Group × Time interaction prevent definitive interpretation of parasympathetic reactivation differences. Nonetheless, tonic GSR, RMSSD, SDNN, and saccade velocity emerge as promising biomarkers for future investigation in larger, well-controlled samples.

## Figures and Tables

**Table 1:** Results of volunteer screening.

	Healthy Control Group (*n* = 46)	OCD Group (*n* = 31)	*p*-Value
Age	27.4 ± 8.6	28.3 ± 7.2	0.621
Sex	28 females/18 males	22 females/9 males	0.467 *
Yale-Brown Obsessive Compulsive Scale (Y-BOCS)	0.4 ± 1.1	21.0 ± 6.4	<0.001
State Trait Anxiety Inventory (STAI)—Trait	41.6 ± 8.0	54.3 ± 11.2	<0.001
State Trait Anxiety Inventory (STAI)—State	33.1 ± 9.3	44.5 ± 11.9	<0.001
Beck Depression Index (BDI)	5.7 ± 4.4	14.8 ± 10.2	<0.001
Barrat Impulsivity Scale (BIS-11)	59.7 ± 8.8	68.8 ± 9.7	<0.001

* Fisher’s exact test (two-tailed *p*-value).

**Table 2:** The results of Spearman correlation analysis between autonomic nervous system (ANS) parameters and assessments of Valence and Arousal.

OCD group	Method	Parameter	Valence		Arousal	
			Spearman R	*p*-Level	Spearman R	*p*-Level
	ECG	MeanRR	−0.216	0.390	−0.050	0.845
		SDNN	−0.061	0.810	−0.389	0.110
		RMSDD	0.168	0.505	**−0.499**	**0.049**
	Eyetracker	Peak Saccade Velocity	−0.342	0.165	0.264	0.289
		Av Saccade Velocity	−0.255	0.307	0.354	0.149
		Av Saccade Amplitude	−0.315	0.203	0.400	0.100
		Av Fix Duration	0.218	0.385	−0.129	0.610
		Pupil size	−0.337	0.171	0.305	0.219
	GSR	Phasic response	0.001	0.997	−0.090	0.723
		Tonic response	−0.117	0.645	0.127	0.616
Control group	Method	Parameter	Valence		Arousal	
			Spearman	*p*-level	Spearman	*p*-level
	ECG	MeanRR	−0.087	0.732	−0.172	0.494
		SDNN	−0.133	0.598	**−0.591**	**0.010**
		RMSDD	0.095	0.708	**−0.487**	**0.050**
	Eyetracker	Peak Saccade Velocity	**0.507**	**0.032**	−0.340	0.168
		Av Saccade Velocity	**0.485**	**0.041**	−0.339	0.169
		Av Saccade Amplitude	**0.510**	**0.031**	−0.321	0.194
		Av Fix Duration	−0.432	0.074	0.119	0.639
		Pupil size	0.395	0.104	−0.303	0.222
	GSR	Phasic response	0.018	0.945	−0.321	0.194
		Tonic response	−0.099	0.696	−0.122	0.630

Uncorrected *p*-values are reported; these analyses were exploratory, and results should be interpreted with caution. Correlations surviving FDR correction (*p* < 0.10) are indicated in bold. Correlational analyses were guided by two a priori hypotheses derived from prior literature: (1) higher arousal is negatively associated with parasympathetic activity (RMSSD, SDNN), and (2) higher valence is positively associated with saccade velocity and amplitude in healthy controls. Uncorrected *p*-values are reported for transparency; these analyses are exploratory, and results should be interpreted cautiously. Bold found was used to mark significant results.

## Data Availability

The data presented in this study are available on reasonable request from the corresponding author.

## Supplementary Materials

The following supporting information can be downloaded at: https://www.mdpi.com/article/10.3390/brainsci16080846/s1. Questionnaire S1. Post-video self-assessment questions. After each video, participants rated (1) perceived pleasantness on a 9-point Likert scale ranging from 1 (very unpleasant) to 9 (very pleasant), and (2) emotional response intensity on a 9-point Likert scale ranging from 1 (no emotion) to 9 (strong emotional response). Figure S1. Schematic summary of the main findings. Individuals with OCD (right) compared to healthy controls (left) showed: (1) higher heart rate (lower mean NN); (2) rising tonic GSR across the experiment; (3) delayed parasympathetic recovery (blunted SDNN/RMSSD increases after positive stimuli); (4) faster saccade velocities during video viewing; and (5) decoupling of saccade velocity from positive valence ratings. Table S1: Demographic and psychotropic medication profile of individuals with obsessive-compulsive disorder (OCD): anonymized, subject-level dataset for 31 individuals diagnosed with OCD. Abbreviations: ID, participant identifier; F, female; M, male; COND, condition; Med, medication status (Yes/No); TCAs, tricyclic antidepressants; SSRIs, selective serotonin reuptake inhibitors; SNRIs, selective serotonin-norepinephrine reuptake inhibitors; SGAs, second-generation antipsychotics; AEDs, antiepileptic drugs. A plus sign (+) indicates active use of the respective drug class; a dash (−) indicates no use. The order of video stimuli and the post-video questionnaire are also provided in the Supplementary File. Table S2. Video stimulus sequence, descriptions, and valence classification. Videos were presented in a fixed pseudorandomized order, with the same sequence for all participants. Each video was 20 s in duration. Valence was determined based on pilot validation ratings.
