# Supplementary material for: Autonomic Dysregulation in Obsessive–Compulsive Disorder: Simultaneous Assessment of Electrodermal, Cardiac, and Oculomotor Responses to Triggering Videos—An Exploratory Study
Source: Brain Sci. 2026 Aug 9;16(8):846. doi: 10.3390/brainsci16080846 (PMC13510467; doi:10.3390/brainsci16080846)
Supplement: Supplementary file 1 [file brainsci-16-00846-s001.zip › brainsci-4364448-supplementary.pdf]

Supplementary Materials

Questionnaire S1. Post-video self-assessment questions. After each video, participants rated (1) perceived pleasantness on a 9-point Likert scale ranging from 1 (very unpleasant) to 9 (very pleasant), and (2) emotional response intensity on a 9-point Likert scale ranging from 1 (no emotion) to 9 (strong emotional response).

1. Please rate how pleasant you find this video on a scale of 1 to 9, where 1 is very unpleasant and 9 is very pleasant.

   very unpleasant 1 - 2 - 3 - 4 - 5 - 6 - 7 - 8 - 9 very pleasant

2. Please rate the strength of your emotional response to this video on a scale of 1 to 9, where 1 indicates no emotion and 9 indicates a strong emotional response.

   weak 1 - 2 - 3 - 4 - 5 - 6 - 7 - 8 - 9 strong

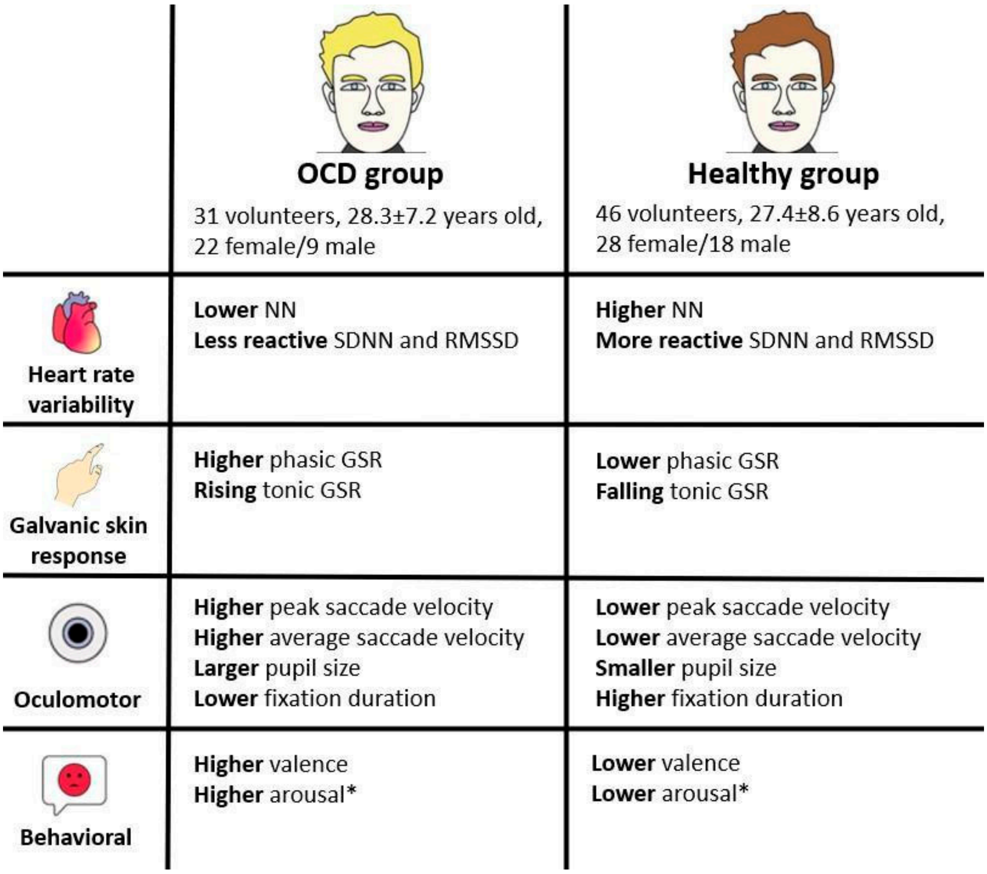

Figure S1. *Schematic summary of the main findings. Individuals with OCD (right) compared to healthy controls (left) showed: (1) higher heart rate (lower mean NN); (2) rising tonic GSR across the experiment; (3) delayed parasympathetic recovery (blunted SDNN/RMSSD increases after positive stimuli); (4) faster saccade velocities during video viewing; and (5) decoupling of saccade velocity from positive valence ratings.*

Table S1: *Demographic and psychotropic medication profile of individuals with obsessive-compulsive disorder (OCD): anonymized, subject-level dataset for 31 individuals diagnosed with*

OCD. Abbreviations: ID, participant identifier; F, female; M, male; COND, condition; Med, medication status (Yes/No); TCAs, tricyclic antidepressants; SSRIs, selective serotonin reuptake inhibitors; SNRIs, selective serotonin-norepinephrine reuptake inhibitors; SGAs, second-generation antipsychotics; AEDs, antiepileptic drugs. A plus sign (+) indicates active use of the respective drug class; a dash (−) indicates no use. The order of video stimuli and the post-video questionnaire are also provided in the supplementary file.

ID	Sex	Age	COND	Med	TCAs	SSRIs	SNRIs	SGAs	AEDs
OCD01	F	22	OCD	No	-	-	-	-	-
OCD02	F	35	OCD	Yes	-	+	-	+	-
OCD03	F	20	OCD	No	-	-	-	-	-
OCD04	F	21	OCD	No	-	-	-	-	-
OCD05	M	33	OCD	Yes	-	+	-	-	-
OCD06	M	40	OCD	No	-	-	-	-	-
OCD07	F	23	OCD	No	-	-	-	-	-
OCD08	F	26	OCD	Yes	-	+	-	-	-
OCD09	F	36	OCD	No	-	-	-	-	-
OCD10	F	30	OCD	Yes	-	-	-	-	+
OCD11	M	37	OCD	No	-	-	-	-	-
OCD12	F	39	OCD	Yes	-	+	-	+	-
OCD13	M	32	OCD	No	-	-	-	-	-
OCD14	F	44	OCD	No	-	-	-	-	-
OCD15	M	28	OCD	No	-	-	-	-	-
OCD16	F	24	OCD	No	-	-	-	-	-
OCD17	F	23	OCD	Yes	-	-	+	-	+
OCD18	M	27	OCD	No	-	-	-	-	-
OCD19	F	24	OCD	Yes	+	-	-	+	-
OCD20	F	23	OCD	No	-	-	-	-	-
OCD21	F	31	OCD	Yes	-	+	-	-	-
OCD22	F	31	OCD	Yes	-	+	-	-	-
OCD23	F	23	OCD	Yes	-	-	+	-	+
OCD24	F	23	OCD	Yes	-	-	+	-	+
OCD25	F	39	OCD	Yes	-	+	-	+	-
OCD26	M	40	OCD	No	-	-	-	-	-
OCD27	F	23	OCD	Yes	-	+	-	+	+
OCD28	M	23	OCD	Yes	-	-	+	-	+
OCD29	F	24	OCD	Yes	+	-	-	+	-
OCD30	F	21	OCD	No	-	-	-	-	-
OCD31	M	33	OCD	Yes	-	+	-	-	-

TCAs - Tricyclic Antidepressants
SSRIs - Selective Serotonin Reuptake Inhibitors
SNRIs - Selective Serotonin-Norepinephrine Reuptake Inhibitors
SGAs — Second-Generation Antipsychotics
AEDs —Antiepileptic Drugs

Table S2. *Video stimulus sequence, descriptions, and valence classification. Videos were presented in a fixed pseudorandomized order, with the same sequence for all participants. Each video was 20 s in duration. Valence was determined based on pilot validation ratings.*

Order	Category	Valence	Description
1	*Checking control 1*	*Positive/Neutral*	*Animated cogs and gears rotate together and slide into place, securely locking and unlocking.*
2	*Aggression OCD 1*	*Negative*	*A woman is threatened by a man in a lift who forces her to undress at knifepoint.*
3	*Checking OCD 1*	*Negative*	*An electric scooter stands in a living room and slowly catches fire from its overheating battery.*
4	*Contamination control 1*	*Positive/Neutral*	*A dirty carpet is gradually cleaned, revealing more and more colour until it is perfectly restored.*
5	*Aggression control 1*	*Positive/Neutral*	*Close-up view as food is thinly sliced by hand with a knife on a chopping board.*
6	*Symmetry OCD 1*	*Negative*	*First-person footage underwater in a swimming pool; hands point to and scrape at mosaic tiles which are asymmetrically placed, breaking the colour pattern.*
7	*Checking control 2*	*Positive/Neutral*	*A house with thick defenses which cover the windows and doors, fitting perfectly over them and blending in with the walls to create a secure block.*
8	*Contamination OCD 1*	*Negative*	*Close-up footage of cockroaches running along walls, doors and surfaces, inside fridges and over used plates.*
9	*Symmetry control 1*	*Positive/Neutral*	*A swimming pool, green with grime, is cleaned to reveal bright blue tiles and a mosaic on the bottom.*
10	*Animal control 1*	*Positive*	*One soft kitten takes up most of the frame, sitting still and looking around. Next, another kitten is shown lying on its back licking its paw.*
11	*Contamination OCD 2*	*Negative*	*First-person footage of a dirty and abandoned underpass filled with litter, old mattresses and cardboard.*

12	*Aggression OCD 2*	*Negative*	*A woman is walking in the forest when she is punched by a man who throws her on the ground and threatens her with a knife.*
13	*Symmetry control 2*	*Positive/Neutral*	*Marbled pottery carving where a circular tool is used to carve away deeper, multicoloured layers of clay, creating a symmetrical pattern.*
14	*Checking OCD 2*	*Negative*	*A dog knocks over an ironing board and iron which falls into a pile of clothes and starts a house fire.*
15	*Aggression control 2*	*Positive/Neutral*	*A knife is used to cut aubergine, tomatoes, chicken and garlic.*
16	*Symmetry OCD 2*	*Negative*	*A circular cake is cut asymmetrically at odd angles before a crooked piece is taken out.*
17	*Contamination control 2*	*Positive/Neutral*	*The inside of a wheel rim is cleaned with high-pressure air, changing colour from black to steel.*
18	*Animal control 2*	*Positive*	*A woman tries to give a cat a bath in the sink while the cat struggles, eventually tearing down the blind.*
